# Urban–rural differences in the relationship between stunting, preschool attendance, home learning support, and school readiness: A study in Côte d'Ivoire

**DOI:** 10.3389/fpubh.2022.1035488

**Published:** 2023-01-09

**Authors:** Abenin Mathieu Brou, Franck Adjé Djalega, Venance Tokpa, Edy Constant Gbala Seri, Apie Léa Fabienne Anoua, Julie Ann Robinson

**Affiliations:** ^1^Institute of Anthropological Development Sciences (ISAD), University of Félix Houphouët-Boigny, Abidjan, Côte d'Ivoire; ^2^Laboratory of Nutrition and Food Security of the Department of Food Science and Technology, University of Nangui Abrogoua, Abidjan, Côte d'Ivoire; ^3^Department of Language Sciences, University of Félix Houphouët-Boigny, Abidjan, Côte d'Ivoire; ^4^Ivorian Center for Studies and Research in Applied Psychology, University of Félix Houphouët-Boigny, Abidjan, Côte d'Ivoire; ^5^Institute of Anthropological Development Sciences (ISAD), University of Félix Houphouët-Boigny, Abidjan, Côte d'Ivoire; ^6^College of Education, Psychology and Social Work, Flinders University, Adelaide, SA, Australia

**Keywords:** stunting, school readiness, urban–rural differences, preschool (kindergarten), home environment, informal learning, low-and middle-income countries, Africa

## Abstract

**Background:**

Stunted physical growth during early childhood is a marker of chronic undernutrition, and the adverse life circumstances that underlie it. These have the potential to disrupt normal brain development and the acquisition of foundational cognitive, language, social and motor skills. Stunting is prevalent in most low-and middle-income countries. Because the prevention of stunting requires large-scale structural and attitudinal changes, several psycho-educational interventions have been developed to mitigate the adverse association between early stunting and skill development. However, the resource-intensive nature of custom-designed interventions limit their sustainability and scalability in resource-limited settings. This study explored the possibility that available resources that promote positive development (existing preschool education programs, and no- or low-cost home-based learning activities and resources) may protect against any negative association between stunting and the acquisition of foundational skills required for academic learning and adaptation at school.

**Method:**

Data for 36-to 59-month-old children (*n* = 3,522; *M* = 46.7 months; 51.2% male; 74.1% rural) were drawn from the most recent Multiple Indicator Cluster Survey conducted in Côte d'Ivoire (MICS5, 2016). Stunting was assessed using the *WHO Child Growth Standards*. Preschool attendance and home learning activities and resources were assessed by maternal report. School readiness was assessed using the 8-item form of the *Early Child Development Index (ECDI)*.

**Results:**

A high percentage of children met the criteria for stunting (28.5%; 19.7% moderate; 8.8% severe). There were marked urban–rural differences in the prevalence of stunting, rates of preschool attendance, home learning activities and resources, children's school readiness scores, and the relationships between stunting, the protective factors and school readiness scores. These urban–rural differences in ECDI scores could be fully explained by differences between these settings in stunting and the protective factors. However, only two protective factors (access to books and home-based activities that promote learning) made independent contributions to variance in ECDI scores. There was tentative evidence that stunted children whose homes provided highly diverse learning activities and multiple types of learning resources were more likely than those who did not to have a high level of school readiness.

**Conclusion:**

Capitalizing on the existing practices of families that show positive deviance in caregiving may provide a basis for culturally appropriate, low-cost interventions to improve school readiness among children in low- and middle-income countries, including children with stunted growth.

## 1. Introduction

Early childhood has a dedicated target in the Sustainable Development Goals. Target 4.2 calls on countries to “ensure that, by 2030, all girls and boys have access to quality early childhood development, care and pre-primary education so that they are ready for primary education” (1). Undernutrition and insufficient informal learning opportunities have been identified as threats to achieving this goal (2).

Diverse adverse life circumstances can result in children experiencing undernutrition. When undernutrition is recurrent or chronic it can lead to stunted linear growth (i.e., height-for-age that is more than two standard deviations below the median height of reference children of the same age and gender) (3). It is estimated that the current prevalence of stunting among children in low- and middle-income countries is 23%, representing over 150 million children (4). Prior to the COVID-19 pandemic, sub-Saharan Africa was the only region in which the number of young children with stunted growth was continuing to increase (5). However, recent projections suggest that the pandemic may have stalled or reversed progress in reducing the prevalence of stunting in several world regions (4).

Stunting during early childhood serves both as an indicator that children's developmental contexts are failing to meet their basic needs, and as a predictor of concurrent and future impaired health, cognitive development, and psychological wellbeing (6). Early childhood is a period in which rapid brain development and the acquisition of foundational cognitive, social, language and motor skills takes place (7). Consequently, when children experience chronic undernutrition and the adverse life circumstances that underlie it during this period, there may be long-term disruptions to their development. For example, research has identified associations between stunting during early childhood and impaired cognitive performance and greater emotional and behavioral problems during adolescence; and a diverse range of health, psychological and employment outcomes during adulthood (8–15).

However, there are a number of gaps in our understanding of the relationship between stunting and the development of foundational skills among young children living in low- and middle-income countries. First, findings are inconsistent. Some research finds associations between stunting and 3- and 4-year-old children's skills in only a subset of low- and middle-income countries [e.g., (16, 17)] and/or for only a subset of foundational skills (17). Moreover, when associations have been found, these often have small effect sizes, regardless of the measures of skills that were used (18, 19). Currently, the factors that contribute to this pattern of findings are poorly understood. Second, most past research has focused on nation-level data. It has rarely explored potential sources of within-country variability, such as urban–rural differences, in associations between stunting and child development. During the past two decades, there have been marked secular trends in the prevalence of stunting in urban and rural settings. These have been influenced by both deteriorating nutrition among the urban poor and improvements in nutrition in many rural areas (20). Consequently, while some recent studies continue to find a lower prevalence of stunting in urban settings [e.g., (18, 21, 22)], others have found no difference, or a rural advantage [e.g., (23, 24)]. Most previous research investigating the association between stunting and children's developmental outcomes has included urban/rural setting as a covariate (18, 19, 25). This has prevented the exploration of setting-specific patterns in the association between stunting and the development of foundational skills during early childhood.

The ultimate goal of national and international initiatives is to prevent stunting. However, due to the diversity of adverse life circumstances that underlie chronic undernutrition, its prevention requires large-scale structural changes that are expensive and cannot be achieved quickly (3, 26). Therefore, a range of interventions with a shorter latency have been developed to mitigate the adverse developmental outcomes associated with stunting during early childhood. These adopt three approaches. The first focuses on improving the adequacy of children's nutrition. Black et al. (27) classify these interventions as nutrition-specific, such as nutritional supplements [e.g., (28)], or nutrition-sensitive, such as water, sanitation and hygiene interventions [e.g., (29)]. The second approach to intervention seeks to address the adverse life circumstances that underlie chronic undernutrition through, for example, conditional cash transfers [e.g., (30)]. The third approach promotes the development of foundational skills among stunted children through parenting [e.g., (11)] or preschool education programs [e.g., (31)]. Ideally, interventions would combine these approaches. Nevertheless, interventions that focus on promoting the development of foundational skills can provide stunted children with long-term protection against adverse cognitive and mental health outcomes [e.g., (15)]. Indeed, a meta-analysis of 75 evaluations of interventions for disadvantaged children under 6 years of age, found that while nutrition interventions produced small positive effects on height-for-age and cognitive, language, and motor skills scores, interventions that provided stimulating activities and/or promoted responsive caregiving produced improvements in these skills that were four to five times greater, even though they had no effect on children's height-for-age (32). Although effective interventions have been identified within each of the three approaches, almost all are resource intensive. This severely limits their sustainability and scalability in most low- and middle-income countries. In addition, most of these interventions import solutions developed in other cultural contexts into the receiving community.

Little research has explored whether existing resources in low- and middle-income countries can be leveraged to protect the development of foundational skills among children who experience stunting during early childhood. The current research addresses this gap. It focuses on three opportunities for learning foundational skills (generic formal preschool programs, and informal low- or no-cost home learning resources and learning activities).

Many international agencies have advocated for a rapid expansion to universal preschool education in low- and middle-income countries (33–36). This has been at least partially inspired by the long-term benefits of preschool programs that were purpose-designed for children raised in poverty in high-income countries [e.g., (37)]. However, it is unclear whether these results will be replicated by generic preschool programs in low- and middle-income countries. These often suffer from poor quality in both structural (e.g., classroom, hygiene, and sanitation facilities; staff:student ratios) and process dimensions (e.g., discipline practices) (38, 39). Although some studies in low- and middle-income countries report a long-term benefit from preschool attendance [e.g., (40)], findings from other studies suggest that many children in these countries achieve very few learning gains (41). For example, in Malawi, even after attending preschool and completing four grades of primary education, children could only identify a mean of 1.29 letters of the alphabet in 1 min (42). At a similar age, typically developing children in the USA (43), and both typically developing children and children with dyslexia in the UK (44), could name the same number of letters in about 1 s.

Informal interactions between young children and their caregivers also often involve activities (e.g., telling stories, playing games) can also promote the acquisition of foundational skills (45, 46). Intervention research in low- and middle-income countries has demonstrated a causal link between caregivers' engagement in these activities and young children's cognitive and socio-emotional development (18). Little research has explored within-country heterogeneity in the frequency or nature of home-based learning activities. However, urban–rural differences in home-based activities that promote learning have been documented in some, but not all, low- and middle-income countries in which this has been examined (47). In addition, there are gaps in our understanding of who engages young children in these activities. Most previous research has focused only on learning activities involving the child's mother or father [e.g., (48)]. This focus is likely to be unhelpful in developmental contexts, such as those in sub-Saharan Africa, where inter-generational households (49), wider kinship involvement in childcare (50) and polygamy (51) can result in children engaging in more learning activities with other adults than with either their mothers or fathers (47, 52).

Young children's skill acquisition can also be promoted through their joint or solo interactions with objects. When this occurs, these objects serve as learning resources. Natural (e.g., sticks, animals, water, sand) and household objects (e.g., cooking pans, adult clothing) provide rich affordances for informal learning. These usually require little or no investment by adults. In contrast, the presence of books and toys in children's homes can serve as an indication of adults' investment in their informal learning. Purpose-designed interventions to promote skill acquisition among stunted children usually include play with toys and book-reading [e.g., (53)]. These two types of learning resources offer different affordances.

Many of the foundational skills young children can acquire in formal and informal learning contexts contribute to their “school readiness”. This is a multi-dimensional concept that encompasses the skills that are “crucial for children's transition and adaptation to school” (54). It includes, but is not limited to, socio-emotional skills, such as emotional regulation and ability to sustain attention; cognitive skills and general knowledge, such as the ability to name the letters of the alphabet and numerals; skills required to learn from classroom lessons, such as a receptive vocabulary sufficient to understand instructions; and physical wellbeing and fine motor skills (55).

**Figure 1:**
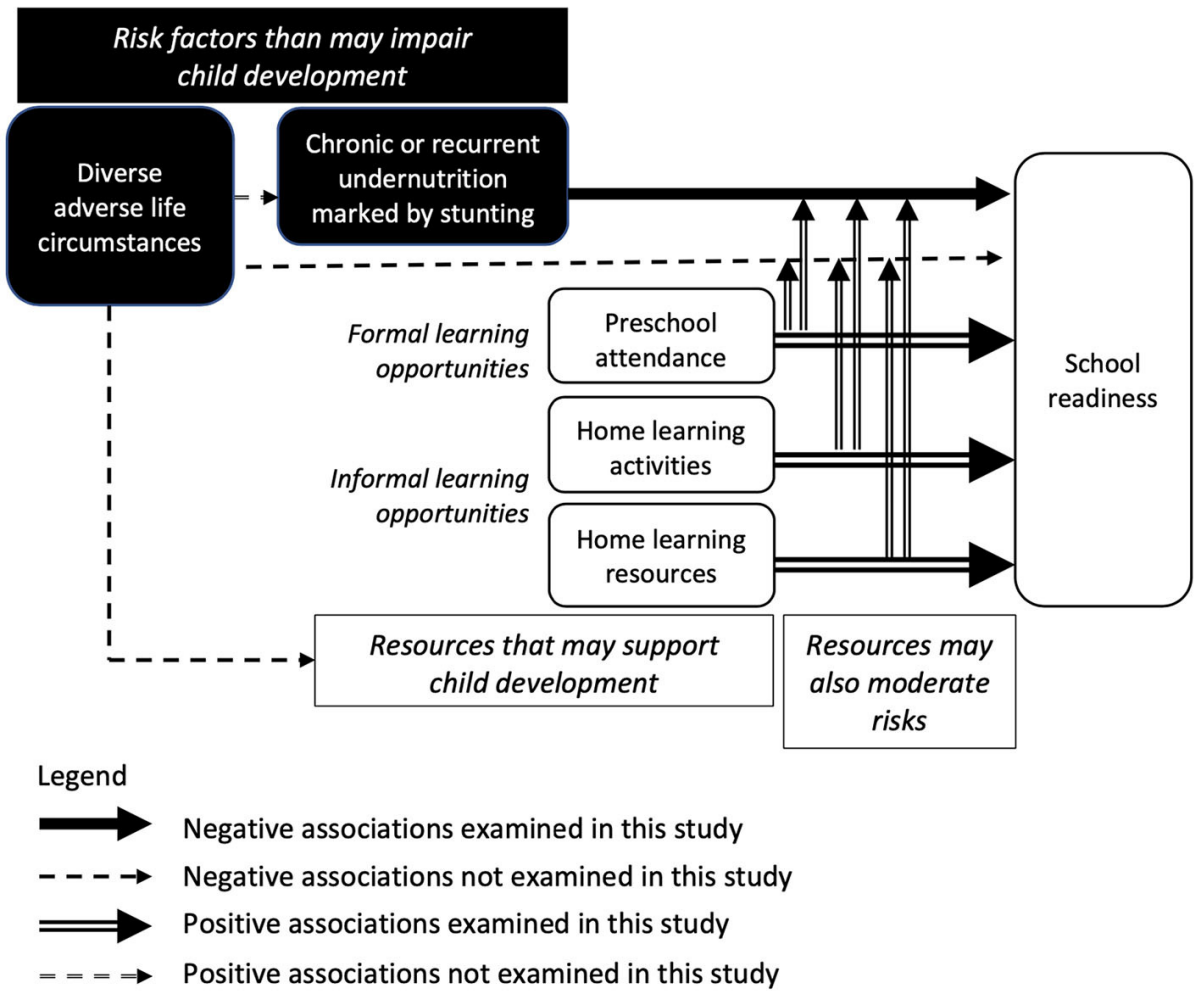
Conceptual model underlying the current research.

In summary, the main aim of the current study is to explore whether school readiness shows a negative linear association with stunting and positive linear associations with three widely available resources that provide opportunities for learning (preschool, and home-based activities and resources that promote learning), and whether these resources also moderate the relationship between stunting and school readiness. Figure 1 shows the conceptual model underlying the current research. Only one previous study appears to have examined the moderating role of preschool attendance and home learning activities on the negative association between stunting and the development of foundational skills among children in low- and middle-income countries (19). It found equivocal results.

Both linear and threshold relationships between the predictor variables and school readiness may provide direction for future interventions. The current research explores the moderated relationships in the conceptual model using both traditional regression analyses to examine linear relationships, and a positive deviance approach to examine threshold relationships (56). A positive deviance approach to intervention is philosophically consistent with research that explores the possibility of leveraging existing community resources to support the development of stunted children. During the 1970s attempts were made to design more respectful and sustainable public health interventions by capitalizing on the positive health behaviors already being practiced by a minority of community members [e.g., (57, 58)], rather than importing interventions developed in other contexts. Such practices showed “positive deviance” from the less beneficial health practices of most members of the community. After the success of this approach in improving children's nutrition [e.g., (59)] and newborn care [e.g., (60)], it has been applied in diverse fields including agriculture [e.g., (61)], medicine [e.g., (62)] and child development [e.g., (63)].

The Republic of Côte d'Ivoire (Ivory Coast) embodies the challenges faced by many low- and middle-income countries and provides a rich context in which to examine the conceptual model. Côte d'Ivoire is a lower-middle-income country in West Africa with a population of about 29 million. Its most recent score on the Human Development Index was very similar to the mean for sub-Saharan Africa and gave it a rank of 162nd out of 188 countries (64). Although it contains two major cities, Yamoussoukro and Abidjan, most of its population lives in rural settings. The average per capital income remains very low, with most of the population earning less than US$3.20 per day (65). However, this average masks large within-country differences. Poverty is far more prevalent in rural (70%) than in urban areas (5.8%) (66). There is a similar rural disadvantage in access to basic amenities that support health. For example, although almost all residents in urban areas have access to safe drinking water and at least basic sanitation facilities, a much lower percentage of rural residents do so (67, 68). In particular, open defecation is used by more than 40% of rural households (36). These elevated health risks are coupled with much lower access to health facilities, and skilled health professionals in rural areas (69). Unsurprisingly, there is also a marked disparity between rural (108 per 1,000) and urban (78 per 1,000) mortality rates among children under 5 years of age (68). Historically, the prevalence of stunting among surviving children under 5 years of age has also been much higher in rural than in urban areas of Côte d'Ivoire (70). Côte d'Ivoire is classified as a “persistent high-burden country” for stunting among young children: the national prevalence rate has remained around 30% for the past two decades (71). Although local production, importation, and food aid are theoretically sufficient for the country's needs (72), the population's access to a healthy, diversified, and nutritious diet remains limited (73).

The population contains about 70 distinct ethnic groups, which can be divided into four broad cultural or sociolinguistic categories (74). Wide cultural and religious diversity is reflected in equally diverse family structures and child-rearing customs. The combined result of widespread polygamy, high fertility (75) and a tradition of multi-generational households is that many children are raised in large households containing several adults. Approximately one-in-three children under 5 years of age lives in a household containing six or more members (75).

Côte d'Ivoire's preschool program also shares the shortcomings of those in many other low- and middle-income countries. Many preschools lack basic infrastructure (77% have no water supply; 43% have no access to electricity) (76), teaching materials (77), and suitably qualified staff (77, 78). In 2016, only approximately one-in-seven children of an eligible age attended an organized preschool program (68). Although there was no gender bias, there were large disparities in preschool attendance associated with mothers' highest level of education and family wealth. However, even when mothers had completed secondary school or higher education fewer than one-in-two of their children attended preschool, and even among the richest families, only about one-in-two children attended (68). The highest rate of preschool attendance was found for children living in the largest city, Abidjan. But even there, fewer than 40% of children who were eligible to attend preschool did so (68). Data concerning Ivoirian parents' motivation for enrolling or not enrolling their children in preschool is lacking.

Because children's exposure to risk factors and resources differs between rural and urban settings in Côte d'Ivoire, the conceptual model will be examined separately in each setting. This analysis plan also addresses the gap in our current knowledge about within-country heterogeneity in the pattern of relationships between stunting, developmental resources and children's acquisition of foundational skills.

The current study addressed six research questions:

**Urban–rural differences:**

1. In comparison with their urban peers, are young children who live in rural settings in Côte d'Ivoire disadvantaged by a higher prevalence of stunting, lower engagement in formal and informal opportunities for learning (preschool attendance, home-based activities that promote learning, home access to books and toys), and lower levels of school readiness?
2. To what extent can urban–rural disparities in school readiness be accounted for by differences between these settings in the prevalence of stunting and opportunities for formal and informal learning?

**Conceptual model:**

3. Does evidence support the assumption in the conceptual model that school readiness is negatively associated with stunting and positively associated with opportunities for formal and informal learning? Are these associations observed in both urban and rural settings?
4. Is any negative linear association between stunting and school readiness moderated by opportunities for formal and informal learning? Is the same pattern of relationships observed in both urban and rural settings?
5. Are stunted children whose scores for opportunities for learning show positive deviance more likely than other stunted children to have acquired adequate school readiness skills?

**Measurement:**

6. To what extent does the inclusion of activities with an adult other than their parents expand the assessment of young children's engagement in home-based activities that promote learning?

## 2. Method

### 2.1. Data source

This research draws on data collected during the latest Multiple Indicator Cluster Survey (MICS5) conducted in Côte d'Ivoire. This survey was designed to monitor progress toward the targets of the Sustainable Development Goals and the National Development Plan for 2016–2020. The survey selected a nationally representative sample of households using cluster sampling in a two-stage probability sampling design. A subsample of 512 of the 23,484 enumeration areas from the *2014 General Population and Housing Census* was selected. Then, a sample of 25 households was recruited in each area. Data were collected between April and July 2016.

In total, 11,879 of the 12,768 selected households responded to the main survey (96.6% participation rate). During data collection, face-to-face interviews were conducted with mothers or primary carers of children under 5 years of age (98.2% participation rate). Trained interviewers recorded the mothers'/caregivers' responses and mothers' and children's anthropometric measurements on tablets.

### 2.2. Participants

MICS5 collected data on 3,730 children aged 36–59 months of age. The planned statistical analyses assume that datapoints are independent. This assumption is violated if a sample includes multiple children born to the same mother and living in the same household. Solutions that involve statistical analyses that cluster children within families face two challenges. First, within-family differences in the parenting practices and the investment of resources directed to individual children (based on their gender, birth order and other factors) are very common. Second, these within-family differences often show little uniformity in multi-cultural populations. Therefore, the current study ensured the independence of data points by randomly selecting one child per mother for inclusion in the analyses. The final sample consisted of 3,522 children (94.4% of the original sample), most of whom (74.1%) lived in rural settings (Table 1).

**Table 1:** Demographic characteristics of the samples of urban and rural 36- to 59-month-old children in Cote d'Ivoire.

	**Urban (*****n*** = **911)**			**Rural (*****n*** = **2,611)**			**Total (*****n*** = **3,522)**		
**Characteristic**	**Mean**	**SD**	**%**	**Mean**	**SD**	**%**	**Mean**	**SD**	**%**
Age (months)	46.9	6.8		46.4	6.7		46.6	6.7	
Male			53.1			50.6			51.2
**Ethnicity**									
Akan			23.7			26.4			25.7
Krou			4.5			5.0			4.9
Mande du Sud			4.8			6.1‘			5.8
Mande du Nord			26.9			14.6			17.7
Gur			17.3			23.9			23.3
Other			22.7			24.9			23.7
**Mothers' education**									
None			56.2			75.9			70.8
Primary			23.1			18.5			19.7
Secondary or higher			20.7			5.6			9.5

### 2.3. Measures

All interviews with mothers/primary caregivers and instructions to children during anthropometric measurements were provided in French or in the participants' local language.

#### 2.3.1. Demographic characteristics

In about 90% of cases, children's age could be accurately determined from their immunization records. When these was not available children's date of birth was reported by the mother/caretaker. Children's sex and ethnicity were assessed by single items.

#### 2.3.2. Children's life circumstances

Data concerning the mothers' education, the number and age of other children, the flooring material of the dwelling, and the household's access to electricity and improved sanitation facilities were gathered through observations by interviewers and/or parent reports. Mothers were asked to indicate the highest level of education they had attended (did not attend primary school = 0; primary school = 1; secondary school or post-secondary education = 2). This wording prevented conclusions about the highest levels of education mothers had completed.

The economic circumstances of households were assessed using a wealth index calculated on the basis of a principal components analysis of data about the ownership of consumer goods, dwelling characteristics, and other characteristics. Separate factor scores were calculated for households in urban and rural areas. Each household in these two samples was then assigned a wealth score based on the assets owned by that household and on the final factor scores. Finally, wealth scores in each setting were ranked and grouped into five categories of equal size (quintiles: poorest = 1; richest = 5). Further information on the construction of the wealth index is provided by Rutstein (79).

*Escherichia coli* (e. coli) contamination of household drinking water at its point-of-use was assessed using samples that were collected by asking for “a glass of water that members of the household would drink”. A sample of 100 ml of water was filtered through a 0.45 micron filter paper that could retain any *E. coli*. The filter was then placed on a growth medium plate. A second sample of 1 ml of water was pipetted directly onto a different growth medium plate. To encourage the growth of *E. coli*, both plates from a given household were stored in custom-designed incubation belts carried by the fieldwork staff, or in electric incubators. After a 24-h period, colonies of *E. coli* (blue) and non-*E. coli* coliforms (purple/red) that were detectable by eye were counted. The risk of fecal contamination in 100 ml of drinking water was classified as low (0 colonies), moderate (1–10 colonies), high (11–100 colonies) or very high (more than 100 colonies) (80).

#### 2.3.3. Urban–rural setting

Children's place of residence was classified as urban or rural on the basis of population size using the criteria from the *2014 General Census of Population and Housing*. Urban and rural settings were sampled in each of the eleven administrative districts in Côte d'Ivoire (except the district for the city of Abidjan, which contains no rural areas).

#### 2.3.4. Stunting

Children's standing height without shoes or socks was measured in accordance with World Health Organization recommendations (81) by trained field assessment teams using a portable stadiometer with an accuracy of 1 mm. Z-scores for height-for-age were interpreted using the *WHO Child Growth Standards* (81). Stunting was classified as moderate (height-for-age z-score under −2, but not under −3) or severe (height-for-age z-score <-3). Z-scores below −5 (*n* = 17) and above +5 (*n* = 1) were removed from the dataset because these are implausible values. The prevalence of stunting was classified according to the thresholds set by the WHO-UNICEF Technical Expert Advisory Group on Nutrition Surveillance: very low (under 2.5%); low (2.5–<10%); medium (10–<20%); high (20–<30%); very high (over 30%) (82).

#### 2.3.5. Preschool attendance

The mother/caretaker was asked whether the child attended a public or private pre-school. Attendance at either type of pre-school was coded as 1, and attendance at neither was coded as 0. Out-of-home childcare without an educational orientation was excluded.

#### 2.3.6. Home-based activities that promote learning

The *Family Care Indicators* inventory (83) asked whether the child's mother, father, or another adult (person over 15 years of age) had engaged in each of six activities with the target child within the previous 3 days: “read books or looked at picture books with your child”; “told stories to your child”; “sang songs with your child”; “took your child outside the home place”; “played games with your child”; and “spent time with your child in naming things or counting things or drawing”. Responses were integrated to assess whether any adult had engaged the child in each of the activities. A composite score was created by summing positive responses across the six activities (range 0–6). The inventory has good reliability, concurrent validity and equivalence across low- and middle-income countries, including Côte d'Ivoire (84). In the current study, the scale had adequate internal consistency in both rural and urban settings (Cronbach alpha of 0.72 and 0.71, respectively). Scores of 4 or more are considered to indicate “adequate” home stimulation (47, 85).

#### 2.3.7. Home-based resources that promote learning

Families' investment in home learning resources was assessed using three questions. The first asked how many children's books (use requires caregivers to be literate) or picture books (use does not require caregivers to be literate) the target child had. The second and third questions were: “I am interested in learning about the things that (name) plays with at home. Does he/she play with home-made toys (such as dolls, cars or other toys made at home)? Toys from a shop or manufactured toys?” Because data on access to books was not expected to have a normal distribution, a dichotomous variable was created (one or more books = 1; no books = 0). Dichotomous data from the two questions about toys were integrated to create a single variable (one or more home-made or manufactured toys =1; no toys = 0).

#### 2.3.8. School readiness

The 3-domain form of the *Early Childhood Development Index* (ECDI) (86) was used as a proxy measure of school readiness. This caregiver-reported checklist consists of dichotomously scored items that assess skills relevant for 3- and 4-year-old children. The 8 items assess literacy and numeracy (3 items; e.g., “Can (name) identify or name at least ten letters of the alphabet?”), social-emotional skills (3 items; e.g., “Does (name) get along well with other children?”) and learning (2 items; “Does (name) follow simple instructions to do something correctly?”). After reverse-scoring the two negatively worded items, the number of positive responses was summed (range 0 to 8). Previous research has also used this 3-domain composite score (87, 88). However, the wide range of child ages, small number of items and dichotomous scoring of items limit the measure's sensitivity (87, 89). For example, children who can name 9 letters and recognize the symbols for 9 numbers receive the same score (0) on these items as children who cannot name any letters or recognize any numbers.

## 3. Results

The large sample sizes resulted in very high statistical power, which had the potential to identify trivial effects. Because of this, and the large number of comparisons, *p* < 0.01 was adopted as the criterion for statistical significance. Group comparisons of ECDI scores were made using ANCOVA that covaried for any differences in child age (months) between the groups.

Statistical analyses were conducted using SPSS 28.0.1.1 (13). Effect sizes are reported as partial eta squared ($\eta_{\text{p}}^{2}$) or Pearson correlation coefficients (r). The effect size for Chi square values was calculated using the *Practical meta-analysis effect size calculator* created by David B. Wilson (https://www.campbellcollaboration.org/escalc/html/EffectSizeCalculator-R5.php).

### 3.1. Preliminary analyses

Ceiling and floor effects in the distribution of data and the potential for collinearity between predictor variables had implications for the main analyses. Therefore, the prevalence of stunting and opportunities for formal and informal learning, ECDI scores, and the inter-relationships between predictor variables were examined in a series of preliminary analyses. These also identified urban–rural differences in the main variables (Research Question 1), described children's developmental contexts, and explored the contribution of different caregivers to home-based learning activities (Research Question 6).

#### 3.1.1. Stunting

##### 3.1.1.1. Prevalence

The overall prevalence of stunting among 36- to 59-month-old children was 28.5% (Moderate: 19.7%; Severe: 8.8%). UNICEF criteria classify the overall prevalence of stunting as high (82). The prevalence of stunting was more than 10 times higher, and the prevalence of severe stunting was more than 60 times higher, than these rates in the reference sample for the *WHO Child Growth Standards* (81). There was no gender difference in the prevalence of moderate (Boys: 21.4%; Girls: 18.0%) or severe stunting (Boys: 8.8%; Girls: 8.7%) ($\chi_{\left(2,\text{N}=3301\right)}^{2}$ = 6.3, ns).

##### 3.1.1.2. Urban–rural differences

The prevalence of moderate and severe stunting was higher in rural than in urban settings (Table 2A). UNICEF criteria classify the overall prevalence of stunting among urban children and rural children as medium and very high, respectively (82).

Table 2Urban–rural differences in the main variables among 36- to 59-month-old children in Côte d'Ivoire.

**Table:** 

**A. Categorical variables**					
**Characteristic**	**Prevalence (%)**		**Statistic**	***p***	**Effect size (** ***r*** **)**
	**Urban (*****n*** = **911)**	**Rural (*****n*** = **2,611)**			
**Stunted linear growth**					
Severe stunting	3.5	10.7	$\chi_{\left(1,N=3,299\right)}^{2}$ = 41.6	**	0.11
Moderate and severe stunting	13.7	33.8	$\chi_{\left(1,N=3,298\right)}^{2}$ = 126.5	**	0.20
Preschool/school attendance	43.8	24.1	$\chi_{\left(1,N=3,521\right)}^{2}$ = 127.4	**	0.19
**Home learning resources**					
One or more children's books	73.4	26.6	$\chi_{\left(1,N=3,520\right)}^{2}$ = 274.5	**	0.28
One or more toys	81.2	55.7	$\chi_{\left(1,N=3,522\right)}^{2}$ = 187.6	**	0.23
**Home learning activities**					
Reading books	29.9	9.3	$\chi_{\left(1,N=3,446\right)}^{2}$ = 224.0	**	0.26
Storytelling	33.5	24.0	$\chi_{\left(1,N=3,447\right)}^{2}$ = 30.7	**	0.09
Singing songs	45.7	35.9	$\chi_{\left(1,N=3,454\right)}^{2}$ = 26.6	**	0.09
Walking outdoors	74.8	63.4	$\chi_{\left(1,N=3,454\right)}^{2}$ = 38.5	**	0.11
Playing games	91.8	85.0	$\chi_{\left(1,N=3,470\right)}^{2}$ = 26.5	**	0.09
Naming, counting, drawing	40.4	16.8	$\chi_{\left(1,N=3,451\right)}^{2}$ = 231.8	**	0.26

**Table:** 

**B. Continuous variables**							
**Characteristic**	**Urban (*****n*** = **911)**		**Rural (*****n*** = **2,611)**		**Statistic**	***p***	**Effect size (** $\eta_{\text{p}}^{2}$ **)**
	**Mean**	**(SD)**	**Mean**	**(SD)**			
**Home learning activities**							
Total score	3.2	(1.7)	2.3	(1.6)	*F*_(1,3,425)_ = 164.3	**	0.046
**Early child development index**							
Literacy and numeracy (0–3)	0.5	(0.5)	0.1	(0.4)	*F*_(1,3,455)_ = 280.9	**	0.075
Learning (0–2)	1.7	(0.6)	1.5	(0.7)	*F*_(1,3,448)_ = 28.4	**	0.008
Socio-emotional skills (0–3)	1.9	(0.9)	2.0	(0.8)	*F*_(1,3,418)_ = 6.6	ns	
Total for all children (0–8)	4.1	(1.5)	3.6	(1.3)	*F*_(1,3,392)_ = 70.0	**	0.020

** *p* < 0.001; ns, not significant; *p* > 0.01.

##### 3.1.1.3. Adverse life circumstances

In both settings, stunting was associated with exposure to multi-dimensional risks to child development (Table 3). In urban settings, the prevalence of each of the eight risk factors that were assessed was higher for children who were stunted than for their peers who were not stunted. In rural settings, four risk factors did not discriminate between children who were and were not stunted. Although the vast majority of stunted children were exposed to three of these risk factors, they were also pervasive among rural children who were not stunted.

**Table 3:** Association between stunting and exposure to risks to child development among 36- to 59-month-old children living in urban and rural settings in Cote d'Ivoire.

	**Prevalence (%)**					
	**Urban**			**Rural**		
**Risk factor**	**Not stunted**	**Stunted**	***p***	**Not stunted**	**Stunted**	***p***
**Low financial and social capital**						
Mother has no formal education	53.3	69.7	***	74.5	75.9	ns
Lowest urban wealth quintile	22.1	48.7	***			
Lowest rural wealth quintile				19.6	24.9	**
**Household lacks basic amenities**						
No electricity supply	8.9	21.8	***	60.3	66.7	**
**Greater health risks**						
High or very high *E. coli* contamination of drinking water^∧^	46.6	73.3	*	85.8	86.0	ns
No access to improved sanitation	30.1	43.7	**	72.0	77.4	**
Dwelling has earth or dung floor^∧^	9.5	29.4	*	91.2	95.2	*
**Caregiving constraints**						
One or more other children under 5 years of age	50.6	64.7	**	66.3	68.8	ns

* *p* < 0.05;

** *p* < 0.01;

*** *p* < 0.001; ns, not significant.

∧ Data for these variables were collected for only ~25% of the sample. Because the analyses for these variables were not over-powered, a criterion for significance of *p* < 0.05 was used.

A criterion of *p* < 0.01 was used in all other analyses.

##### 3.1.1.4. Association with other predictor variables

In urban, but not in rural settings, preschool attendance rates and the diversity of home-based activities that promote learning were lower for children with stunting than without stunting (Table 4). In contrast, both urban and rural settings showed the same pattern of results for home learning resources: stunted children were less likely to have one or more children's books or picture books, but equally likely to have one or more purchased or home-made toys, when compared with their peers who were not stunted (Table 4). All effect sizes were small.

**Table 4:** Formal and informal learning opportunities for 36- to 60-month-old children in Côte d'Ivoire who were and were not stunted.

	**Not stunted**			**Stunted**			**Statistic**	***p***	**Effect size**
	**M**	**(SD)**	**%**	**M**	**(SD)**	**%**			
**Preschool attendance**									
Urban			46.7			30.3	$\chi_{\left(1,N=868\right)}^{2}$ = 11.3	**	*r* = 0.11
Rural			25.5			24.1	$\chi_{\left(1,N=3,521\right)}^{2}$ = 0.6	ns	
**Home learning resources**									
**One or more books**									
Urban			19.4			7.6	$\chi_{\left(1,N=867\right)}^{2}$ = 9.8	*	*r* = 0.11
Rural			3.0			0.9	$\chi_{\left(1,N=2,429\right)}^{2}$ = 11.1	**	*r* = 0.07
**One or more toys**									
Urban			82.3			74.8	$\chi_{\left(1,N=869\right)}^{2}$ = 3.8	ns	
Rural			58.7			54.3	$\chi_{\left(1,N=2,429\right)}^{2}$ = 4.4	ns	
**Home learning activities (0–6**)									
Urban	3.3	(1.7)		2.5	(1.6)		*F*_(1,841)_ = 22.7	**	$\eta_{\text{p}}^{2}$ = 0.026
Rural	2.4	(1.6)		2.3	(1.6)		*F*_(1,2,362)_ = 3.9	ns	

* *p* < 0.01;

** *p* < 0.001; ns, not significant; *p* > 0.01.

#### 3.1.2. Formal opportunities for learning

##### 3.1.2.1. Prevalence and urban rural differences

The overall rate of preschool attendance was low (29.2%). This was not due to the inclusion of 3-year-old children in the sample. There was no difference between 3-year-old and 4-year-old children in the rate of preschool attendance in either rural or urban settings (Table 5). However, the rate in urban settings was almost twice that in rural settings (Table 2A).

**Table 5:** Differences in preschool attendance and school readiness scores between 3- and 4-year-old children in Côte d'Ivoire.

	**3-years-old**			**4-years-old**			**Statistic**	***p***	**Effect size**
	**M**	**(SD)**	**%**	**M**	**(SD)**	**%**			
**Preschool attendance**									
Urban			43.3			55.6	$\chi_{\left(1,N=910\right)}^{2}$ = 0.1	ns	
Rural			24.5			23.5	$\chi_{\left(1,N=2,611\right)}^{2}$ = 0.3	ns	
**School readiness** ^∧^									
Urban	3.8	(1.2)		4.2	(1.5)		F_(1,877)_ = 18.6	**	$\eta_{\text{p}}^{2}$ = 0.021
Rural	3.5	(1.2)		3.9	(1.2)		F_(1,2,514)_ = 55.3	**	$\eta_{\text{p}}^{2}$ = 0.022

^**^p < 0.001; ns, not significant, *p* > 0.01.

^∧^Total score on three-domain form of the Early Child Development Index (possible range: 0–8).

##### 3.1.2.2. Association with other predictor variables

In both urban and rural settings, children who attended preschool also engaged in more diverse learning activities at home (Table 6). Similarly, in both setting, children who attended preschool were more likely than their peers to have one or more books and one or more manufactured or home-made toys (Table 6). Although the effect size for most associations was small, in urban settings, associations between preschool attendance and home learning activities and book ownership were large and moderate, respectively. Collinearity would be introduced into the main analyses if preschool attendance and these variables were included.

**Table 6:** Home learning resources and activities for 36- to 59-month-old children in Côte d'Ivoire who did and did not attend preschool.

	**Attended preschool**			**Did not attend preschool**			**Statistic**	***p***	**Effect size**
	**M**	**(SD)**	**%**	**M**	**(SD)**	**%**			
**Home learning activities**									
Urban	3.9	(1.6)		2.6	(1.6)		F_(1,882)_ = 161.8	**	$\eta_{\text{p}}^{2}$ = 0.155
Rural	2.8	(1.6)		2.2	(1.6)		F_(1,2,541)_ = 73.4	**	$\eta_{\text{p}}^{2}$ = 0.028
**Home learning resources**									
**Book(s)**									
Urban			32.7			5.7	$\chi_{\left(1,N=908\right)}^{2}$ = 113.3	**	*r* = 0.35
Rural			6.0			1.0	$\chi_{\left(1,N=2,611\right)}^{2}$ = 55.6	**	*r* = 0.15
**Toy(s)**									
Urban			90.5			74.0	$\chi_{\left(1,N=908\right)}^{2}$ = 40.0	**	*r* = 0.21
Rural			64.2			53.0	$\chi_{\left(1,N=2,611\right)}^{2}$ = 24.5	**	*r* = 0.10

** *p* < 0.001; ns, not significant, *p* > 0.01.

#### 3.1.3. Informal opportunities for learning

Overall, very few children (6.2%) had access to one or more children's books or picture books at home, but most children (62.3%) had at least one home-made or manufactured toy. Both resources were more accessible in urban than in rural settings, although the effect size was small (Table 2A).

The diversity of activities in which adults engaged young children was also low (M = 2.5, SD = 1.7, possible range: 0–6). This diversity was higher in urban settings than in rural settings, although, again, the effect size was small (Table 2B). *Post-hoc* analyses found that all the assessed learning activities were more common in urban than in rural settings (Table 2A). Two activities (reading books or looking at picture books, and naming, counting or drawing with the child) were more than twice as common in urban than in rural settings. Despite this, even in urban settings adults engaged fewer than one-in-three children in interactions with books and fewer than one-in-two children in activities that involved naming, counting or drawing.

##### 3.1.3.1. Caregivers involved in home learning activities

The planned analysis addressing Research Question 6 explored the frequency with which mothers, fathers and other adults within the household had recently engaged the child in activities that promote learning (Table 7). Many children had participated in one or more of these activities only with adults other than their parents. Consequently, scores for these children would have been even lower if the assessment of home learning activities had focused only on parents. However, even when their scores included activities with other adults, few children in either rural (23%) or urban settings (41%) received adequate home stimulation by the criteria established in previous research in low- and middle-income countries (85).

**Table 7:** Prevalence with which different caregivers engaged urban and rural 36- to 59-month-old children in Côte d'Ivoire in home-based activities that promote learning.

	**Prevalence (%)**	
**Activity and caregiver**	**Urban (*****n*** = **910)**	**Rural (*****n*** = **2,600)**
**Read book**		
Mother	13.1	3.1
Father	8.0	2.4
Other adult	17.3	5.2
**Told story**		
Mother	20.0	15.6
Father	10.0	8.2
Other adult	10.8	7.2
**Sang songs**		
Mother	30.7	24.9
Father	7.6	6.2
Other adult	18.4	13.2
**Went for a walk**		
Mother	52.2	42.1
Father	22.9	23.2
Other adult	30.4	30.5
**Played games**		
Mother	52.7	39.2
Father	28.4	23.3
Other adult	61.1	65.2
**Named, counted or drew**		
Mother	19.6	6.3
Father	11.1	5.2
Other adult	21.9	9.3

##### 3.1.3.2. Association with other predictor variables

In both urban and rural settings, children who had one or more children's books or picture books, or had one or more home-made or manufactured toys, also engaged in more diverse home-based learning activities than their peers (Books: Urban: F_(1,881)_ = 181.5, *p* < 0.001, $\eta_{\text{p}}^{2}$ = 0.171; Rural: F_(1,1,254)_ = 107.3, *p* < 0.001, $\eta_{\text{p}}^{2}$ = 0.041; Toys: Urban: F_(1,883)_ = 81.6, *p* < 0.001, $\eta_{\text{p}}^{2}$ = 0.085; Rural: F_(1,1,241)_ = 87.6, *p* < 0.001, $\eta_{\text{p}}^{2}$ = 0.034;). In urban settings, the effect sizes for books and toys were large and medium, respectively. Collinearity would be introduced into the main analyses if home based activities, and book and toy ownership were included.

#### 3.1.4. School readiness

Overall, the mean total score on the ECDI represented achievement of less than half of the items assessing skills relevant to school readiness (M = 3.7, SD = 1.3). The inclusion of 3-year-old children in the sample can only partially account for this. Although ECDI scores for 4-year-old children were higher than those for 3-year-old children in both rural and urban settings, the effect sizes were small (Table 5). Even 4-year-old children living in urban settings had achieved only about half of the assessed skills.

##### 3.1.4.1. Urban–rural differences

Children living in urban settings had higher total ECDI scores than those in rural settings (Table 2B). *Post-hoc* analyses found that urban children had higher scores for both learning and literacy and numeracy subscale scores. While the effect size for learning was very small, the effect size for literacy and numeracy was large (Table 2B).

#### 3.1.5. Summary

Research Question 1 addressed urban rural differences in children's developmental contexts and their school readiness. Although the effect sizes were small, children living in rural settings were more disadvantaged than their urban peers in all of the key variables: they had a higher prevalence of stunting, lower rates of preschool attendance, engaged in less diverse home learning activities, had fewer types of home learning resources, and achieved lower school readiness scores. Their life circumstances also gave them high exposure to many risks to positive development (Table 3).

Consistent with the conceptual model, there was evidence that exposure to a diverse range of developmental risks was associated with stunting during early childhood. Research Question 6 explored which household members engaged children in learning activities. Adults other than the child's mother or father engaged children in all the activities that were assessed. Other adults engaged children in most activities with a prevalence that was comparable to that of mothers, and approximately twice as high as that of fathers. A number of inter-relationships between predictor variables showed medium-to-strong effect sizes. Consequently, if all the variables in the conceptual model were entered into the main analysis, collinearity would violate the assumptions underlying the statistical tests.

### 3.2. Conceptual model

#### 3.2.1. Direct linear relationships between individual predictor variables and ECDI scores

Research Question 3 addressed the key assumption underlying the conceptual model: that each of the predictor variables was individually associated with ECDI scores.

##### 3.2.1.1. Stunted linear growth

ANCOVA analyses compared the ECDI scores of children who were and were not stunted. In rural settings, there was no difference between these groups' ECDI scores (Table 8). In contrast, in urban settings, stunted children had lower ECDI scores than children who were not stunted, although the effect size was small (Table 8). There was no evidence in either urban or rural settings that children with severe stunting had lower ECDI scores than children with moderate stunting (Urban: Moderate: 3.5, SD = 1.2; Severe: 3.8, SD = 1.5; Rural: Moderate: M = 3.6, SD = 1.2; Severe: M = 3.6, SD = 1.2).

**Table 8:** Association between individual predictor variables and Early Child Development Index scores among urban and rural 36- to 59-month-old children in Côte d'Ivoire.

	**Total early child development index score (0–8)**									
	**Urban**					**Rural**				
**Predictor variable**	**M**	**(SD)**	**Statistic**	***p***	$\eta_{\text{p}}^{2}$	**M**	**(SD)**	**Statistic**	***p***	$\eta_{\text{p}}^{2}$
Linear growth			*F*_(1,837)_ = 13.2	**	0.016			*F*_(1,2,339)_ = 1.1	ns	
Not stunted	4.2	(1.5)				3.7	(1.3)			
Stunted	3.6	(1.3)				3.6	(1.3)			
Preschool			*F*_(1,875)_ = 36.1	**	0.040			*F*_(1,2,513)_ = 5.5	ns	
Attended	4.4	(1.6)				3.7	(1.2)			
Did not attend	3.8	(1.3)				3.6	(1.2)			
Learning resources										
Books			*F*_(1,876)_ = 92.8	**	0.096			*F*_(1,2,513)_ = 84.3	**	0.032
One or more	5.1	(1.7)				5.2	(1.5)			
None	3.9	(1.3)				3.6	(1.2)			
Toys			*F*_(1,876)_ = 16.0	**	0.018			*F*_(1,2,513)_ = 11.0	**	0.004
One or more	4.2	(1.5)				3.7	(1.2)			
None	3.7	(1.4)				3.5	(1.2)			
Diversity of home learning activities			*r*_(878)_ = 0.152	**				*r*_(2,553)_ = 0.087	**	

** *p* < 0.001; ns, not significant; *p* > 0.01.

##### 3.2.1.2. Preschool attendance

In urban, but not in rural settings, children who attended preschool had higher ECDI scores than children who did not. The effect size in urban settings was small (Table 8).

##### 3.2.1.3. Home learning activities and resources

In both urban and rural settings, children with access to books and toys had higher ECDI scores than those who did not (Table 8). For books, the difference in urban settings was moderate. All other effect sizes were small. In both urban and rural settings, there was also a positive correlation between the diversity of learning activities in which children engaged and their ECDI scores. Both effect sizes were small (Table 8).

##### 3.2.1.4. Summary

The direct linear relationships between individual predictor variables and ECDI scores underlying the conceptual model were found only for children living in urban settings. In rural settings, ECDI scores showed no negative association with stunting and no positive association with preschool attendance.

#### 3.2.2. Predictor variables contributing independent variance to ECDI scores

The conceptual model also assumed that each of the predictor variables contributed independent variance to ECDI scores. The planned linear regression analyses examination of the full model in urban settings was precluded by the medium to strong associations found between home learning activities and both preschool attendance and home learning resources. Because the home learning activities measure produced a range of scores, it was better suited to regression analyses than the dichotomous measures. Therefore, collinearity was avoided by deleting preschool attendance and home learning resources from regression analyses for data from urban settings.

The results of the hierarchical linear regression analysis for data from urban settings are summarized in Table 9. Step 1 showed that the control variable, child age, accounted for 2.5% of the variance in ECDI scores. Adding the two variables from the conceptual model (stunting and home learning activities) explained an additional 8.1% of the variance in ECDI scores. However, this increase was solely due to home learning activities. Stunting status did not contribute independent variance to ECDI scores.

**Table 9:** Summary of findings from hierarchical regression analyses for the relationship between predictor variables and *Early Child Development Index* scores for 36- to 60-month old children living in urban and rural settings in Côte d'Ivoire.

	**Step 1**				**Step 2**			
**Setting and variables**	**B**	**SE B**	***ß***	***p***	**B**	**SE B**	***ß***	***p***
**Urban setting**								
Child age (months)	0.035	0.008	0.161	^**^	0.032	0.007	0.147	^**^
Stunting status					−0.355	0.142	−2.50	ns
Home learning activities					0.224	0.029	0.260	^**^
Step 1: Adjusted R^2^ = 0.025								
F_(1,822)_ = 21.9^**^								
Step 2: R^2^ change = 0.81								
F_(2,820)_ = 37.4^**^								
**Rural setting**								
Child age (months)	0.032	0.004	0.158	^**^	0.030	0.004	0.158	^**^
Stunting status					−0.025	0.054	−0.473	ns
Preschool attendance					−0.020	0.060	−0.007	ns
Home learning activities					0.025	0.017	0.032	ns
One or more books					1.352	0.174	0.162	^**^
One or more toys					0.102	0.052	0.041	ns
Step 1: Adjusted R^2^ = 0.028								
*F*_(1,2,312)_ = 66.7^**^								
Step 2: R^2^ change = 0.034								
*F*_(2,2,307)_ = 16.5^**^								

** *p* < 0.001; ns, not significant; *p* > 0.01.

In data for rural children, child age accounted for a similar percentage (2.8%) of the variance in ECDI scores. Adding the five variables from the conceptual model (stunting, preschool attendance, home-based learning activities, books and toys) explained only an additional 3.4% of the variance in ECDI scores. This increase was solely due to book ownership. None of the other variables in the conceptual model contributed independent variance to ECDI scores.

#### 3.2.3. Moderation: Indirect associations between formal and informal learning opportunities and ECDI scores

##### 3.2.3.1. Linear relationships

The data did not support the analyses necessary to answer Research Question 4. It was not possible to examine whether preschool attendance or home learning activities or resources moderated the linear association between stunting and ECDI scores because there was no association between stunting and ECDI scores in either rural or urban settings once other predictors were entered into analyses.

##### 3.2.3.2. Positive deviance

A positive deviance approach focuses on threshold rather than linear relationships between predictor and outcome variables. Only two variables, home-based learning activities and home-based learning resources allowed cases of positive deviance to be identified. The small minority of households in which adults engaged the child in all, or all but one, of the assessed home learning activities (14.1%), and the even smaller minority of households that provided one or more books and one or more toys (5.8%), were judged to demonstrate positive deviance for home learning activities and home learning resources, respectively. Chi square analyses explored whether stunted children whose home activities or resources showed positive deviance were more likely to have ECDI scores that reflected an adequate level of skills for school entry (>6, i.e., 75% of items including at least 1 pre-numeracy or pre-literacy skill). The results of these analyses should be interpreted with caution due to the low number of children who met the criterion for high ECDI scores. This also precluded the possibility of conducting separate analyses for urban and rural settings.

Stunted children with high home learning activity scores were more likely than other stunted children to have high ECDI scores (Stunted, high home activities score: 6.5%; Stunted, not high home activities scores: 0.5%; $\chi_{\left(1,\text{N}=900\right)}^{2}$ = 27.3, *p* < 0.001, *r* = 0.17). However, this pattern was not unique to stunted children. Children who were not stunted and had high home learning activity scores were also more likely than other children without stunting to score highly on the ECDI (Not stunted, high home activities score: 23.5%; Not stunted, not high home activities scores: 3.9%; $\chi_{\left(1,\text{N}=2,239\right)}^{2}$ = 176.3, *p* < 0.001, *r* = 0.28).

Similarly, stunted children in households that provided both book and toy learning resources were more likely than other stunted children to have high ECDI scores (Stunted, high home learning resources: 31.3%; Stunted, not high home learning resources: 0.9%; $\chi_{\left(1,\text{N}=925\right)}^{2}$ = 104.7, *p* < 0.001, *r* = 0.34). Again, this pattern was not unique to stunted children. Children who were not stunted and had high home learning resources were also more likely than other children without stunting to score highly on the ECDI (Not stunted, high home activities score: 42.9%; Not stunted, not high home activities scores: 3.9%; $\chi_{\left(1,\text{N}=2,275\right)}^{2}$ = 188.0, *p* < 0.001, *r* = 0.29).

These findings provide tentative evidence that when stunted children engage in highly diverse home-based activities that promote learning, or when they had both book and toy learning resources, they were more likely than other stunted children to have high ECDI scores.

### 3.3. Accounting for urban–rural differences in school readiness scores

Urban/rural setting is a distal predictor of children's outcomes; stunting and home learning resources and activities are more proximal predictors. Research Question 2 concerned the extent to which urban–rural differences in ECDI scores could be explained by differences between these settings in children's exposure to risk (stunting) and resource proximal variables (preschool attendance, home activities that promote learning, home access to books and toys). An ANCOVA compared urban and rural children's ECDI scores when child age and the proximal predictor variables were included as covariates. As reported earlier (Table 2), when only children's age was included as a covariate, there was a small urban–rural difference in ECDI scores. However, when the proximal predictor variables were added as covariates, there was no longer a difference between ECDI scores for children in urban and rural settings (F_(1,3,127)_ = 5.3, ns). That is, differential exposure to the proximal predictor variables fully accounted for the urban–rural difference in children's ECDI scores. However, only child age and two of the proximal predictor variables showed between-subject effects (Age: F_(1,3,127)_ = 73.5, *p* < 0.001, $\eta_{\text{p}}^{2}$ = 0.023; Home-based activities that promote learning: F_(1,3,127)_ = 13.3, *p* < 0.001, $\eta_{\text{p}}^{2}$ = 0.004; Children's or picture books: 115.1, *p* < 0.001, $\eta_{\text{p}}^{2}$ = 0.036).

To facilitate comparison with the results from previous research, effect sizes for home learning activities and book ownership were calculated as mean standard difference values (d). The difference between the ECDI scores of children who engaged in all or all but one of the home learning activities and those who did not was 0.66 standard deviations [95% CI 0.50–0.81] in urban settings and 0.32 standard deviations [95% CI 0.19–0.45] in rural settings. The difference between the ECDI scores of children who had one or more children's or picture books and those who did not was 0.85 standard deviations [95% CI 0.67–1.03] in urban settings and 1.25 standard deviations [95% CI 0.98–1.52] in rural settings.

## 4. Discussion

The main goal of the current study was to explore the plausibility of designing interventions for stunted children that leverage existing resources in low- and middle-income countries. To do this, the study examined the relationship between young children's stunting status, their opportunities for formal and informal learning, and a subset of the skills they need to make a successful transition to school. In acknowledgment of the differences between the developmental contexts of children living in urban and rural settings, these relationships were examined separately in each setting.

### 4.1. Stunting

This study confirmed the high burden of stunting borne by many countries in sub-Saharan Africa.

It found that almost 30% of children between 36- and 59-months-of-age in Côte d'Ivoire had stunted linear growth. This is consistent with reports from neighboring countries, including Benin [32%, (90)], Cameroon [31.7%, (91)] and Guinea Conakry [32.4%, (92)]. One of the determinants of stunting is food insecurity (93). In 2017, this too had a rate of about 30% across sub-Saharan Africa (94).

However, national estimates of the prevalence of stunting in early childhood can obscure marked disparities within populations. In this study, the prevalence of stunting in rural areas was almost three times higher than in urban centers. Stunting was also associated with life circumstances that exposed children to a diverse range of developmental risks. Urban–rural differences in stunting are likely to be a consequence of higher exposure to such risks due to persistent rural socio-economic disadvantage, reflected in low family income, poor education, poor housing, and inadequate access to food, clean water, and health services (95, 96).

Stunting is an indication of chronic undernutrition, and the adverse life circumstances that underlie it. Both can disrupt brain development and result in a cascade of disruptions to the development of foundational skills. There is ample evidence of a cause-effect relationship between undernutrition and brain development. For example, infants hospitalized for kwashiorkor, a severe form of undernutrition, show structural disruptions to brain development that can be reversed by timely and appropriate nutrition interventions [e.g., (97)].

However, the current study raises questions about the association between undernutrition marked by stunting and the development of foundational skills among community-dwelling children. It found no evidence that 3- and 4-year-old children with severe stunting had lower ECDI scores than children with moderate stunting, and no evidence that children's stunting status was associated with their ECDI scores beyond that which was attributable to the association between stunting and other predictor variables. These results may be an artifact of the low school readiness scores achieved by most children in the current sample and/or the limited sensitivity of the measure of school readiness, or the effects of chronic malnutrition may not be obvious until later ages when children need to learn more complex skills. However, the current results fit within a wider pattern of findings. Previous research has also failed to find an association between stunting and 3- and 4-year-old children's foundational skills in many other low- and middle-income countries, and when it was found, it usually had only a small effect size [e.g., (17, 18)]. This was the case even when research used more detailed measures of skill acquisition that were adapted to the local context [e.g., (19)]. Moreover, results of a systematic review (98) and meta-analysis (32) found that interventions that combined support for skill development and nutrition were rarely more effective in improving children's foundational skills than interventions that provided only support for skill development, and that interventions that were effective in increasing height-for-age often had little or no effect on the development of children's foundational skills. That is, neither observational nor experimental research findings are consistent with a cause-effect relationship between chronic or recurrent undernutrition, indicated by stunting, and the development of foundational skills in community-dwelling children. The evidence is more consistent with the interpretation that adverse life circumstances both impair the development of children's foundational skills and contribute to undernutrition resulting in stunting. When framed by this interpretation, the current findings suggest that a latent variable, adverse life circumstances, was associated with both undernutrition (marked by stunting) and limited opportunities for informal learning (marked by low diversity in home learning activities and a lack of access to children's books and picture books). School readiness was associated only with the markers of opportunities for informal learning.

Such an interpretation does not diminish the importance of stunting as a focus for public health initiatives. Chronic malnutrition has adverse effects on mortality, health and physical development across childhood, and no research suggests that it is positively associated with the development of foundational skills, academic achievement or mental health. However, when the goal of interventions for stunted children includes supporting their development of foundational skills, an exclusive focus on improving their nutrition may be misplaced.

### 4.2. Preschool attendance

Although the national rate of preschool attendance by 3- and 4-year-old children in Côte d'Ivoire was low (29.2%), this is comparable to the rates for children of the same ages in other low- and middle-income countries (33.6%: 85; 33.1%: 86).

The preschool attendance rate was almost twice as high in urban as in rural areas. This is also consistent with previous research. The absolute magnitude of this rural disadvantage (−20%) is very similar to the mean rural-urban difference across 25 sub-Saharan African countries (−19%) (85). Challenges in providing high quality preschool services in rural settings are common across sub-Saharan Africa (99), and geographic and financial barriers may prevent children from attending those that do exist (100). One small study suggests that the cost of the required materials is one of the main reasons that parents in rural Côte d'Ivoire do not send their children to preschool (101).

However, in contrast to most previous research, this study found little evidence that preschool attendance was associated with higher ECDI scores. Earlier research reported a moderate positive association between preschool attendance and scores on this measure for children in 28 low- and middle-income countries, even after access to books and home learning activities were included in the analysis (102). The authors explained that the strength of this association was partly an artifact, “because the items on the ECDI… reflect skills that are explicitly taught in ECE [early childhood education] centers” (p. 271). The current study's failure to find a meaningful association between preschool attendance and ECDI scores despite this artifact suggests that, in 2016, Côte d'Ivoire could have been added to the list of African countries in which children made few learning gains while attending preschool (41).

### 4.3. Home-based activities that promote learning

Few rural (27%) or urban (41%) children in Côte d'Ivoire met the criteria for sufficiently diverse home learning activities. The magnitude of the rural disadvantage (−14%) is consistent with that reported for neighboring countries (103), and the mean across 25 sub-Saharan African countries (−12%) (85). However, the absolute magnitude of both figures contrasts sharply with comparable data. Two multi-national studies using the same measure and the same age group reported that on average about 70% of children in low- and middle-income countries met this criterion (85, 104). The contrast is even more striking when the criterion for high levels of diversity is applied. Although 57% of children across 63 low- and middle-income countries met this criterion (104), only 14% of children in the current study did so. However, the current findings are consistent with earlier reports that Côte d'Ivoire had the third-lowest score for home-based learning activities among the 25 African countries in which this was examined (85).

In both urban and rural settings, there was a small positive association between the diversity of home activities and the measure of school readiness. This is consistent with two previous studies based on different sets of low- and middle-income countries (19, 87).

The current findings also inform measurement strategies for assessing young children's engagement in home learning activities. Many children only engaged in a specific activity with adults other than their parents. Thus, the focus on interactions with parents that characterizes much previous research will under-represent the diversity of these children's opportunities for learning. Households comprising extended families continue to be common in many low- and middle-income countries, allowing regular contributions to childcare from a wide variety of adults, including grandparents and other wives [e.g., (105)]. Future research could consider also including children's engagement in informal learning activities with sibling caregivers, who make a significant and valued contribution to childcare in many low- and middle-income countries [e.g., (106)].

### 4.4. Home-based resources that promote learning

Most children in both urban and rural settings had one or more manufactured or home-made toys, but very few children in either setting had access to children's books or picture books. This pattern is consistent with findings from neighboring countries (103) and low- and middle-income countries elsewhere in the world (87, 107). Simple home-made toys can be created or adapted from natural materials, household objects or other no- or low-cost items, and their use rarely requires an educated play partner. In contrast, books (or the materials to create books) need to be purchased. It is unclear whether the children in the current study owned picture books or books containing text. However, more than half the mothers in urban settings and more than three-quarters of the mothers in rural settings had no formal schooling, and were therefore unlikely to be able to read books containing text to their children. Consequently, responses to the book-reading item on the measure of learning activities indicated that children were more likely to be read to by other members of the household than by their parents.

### 4.5. School readiness

The average ECDI score was low, representing fewer than half of the assessed school readiness skills. Moreover, in both urban and rural settings, there was only a small difference (representing less than half of one item) between ECDI scores for 3- and 4-year-old children. Although urban children had higher total ECDI scores than rural children, the magnitude of this difference was small, and was fully accounted for by differences between the settings in the prevalence of stunting and children's formal and informal opportunities for learning (although only access to children's books or picture books and home-based activities that promote learning made independent contributions).

It was not possible to examine whether any linear relationship between stunting status and school readiness was moderated by children's opportunities for learning, because no independent linear relationship between stunting and ECDI scores was found in either rural or urban settings. However, tentative support for such a moderating role was provided by a positive deviance approach that explored threshold relationships. Stunted children were more likely to achieve a high school readiness score if they had opportunities to engage in highly diverse home learning activities, or if they had one or more books and one or more toys. Before policy implications can be drawn from these findings, they would need to be confirmed in research in which a larger sample of children attained high school readiness scores.

### 4.6. Interventions

Because the ECDI has not been used as an outcome variable in evaluations of interventions delivered to young children with stunted growth, it is not possible to directly compare the effect sizes observed in the current study with those achieved by these interventions. Moreover, most interventions delivered to stunted children have focused on children under 2 years of age; evaluated outcomes for a pooled sample of children with stunted growth and other children living in adverse life circumstances; or focused exclusively on growth, health and/or motor development outcomes. However, two systematic reviews of skill development, nutrition and combined interventions provide points of reference. Grantham-McGregor et al. (98) reported effect sizes for short-term outcomes from combined interventions delivered at child ages that overlapped those in the current sample. These ranged between a detriment of −0.45 to −0.35 standard deviations (for memory and language skills following two “preschool plus meals” programs) to a benefit of 1.13 standard deviations (for language skills following a three-year intensive multi-sectorial intervention). Prado et al. (32) did not review skill development interventions within the relevant age range but reported mean effect sizes between 0.40 and 0.50 standard deviations for cognitive, language and motor skills when these interventions were delivered at other ages. In the current study, the effect sizes for highly diverse home learning activities and ownership of one or more children's or picture books were equal to, or larger than, the effect sizes for many of the interventions included in these two reviews. The effect size for the association between highly diverse home learning activities and ECDI scores was 0.66 and 0.32 standard deviations in urban and rural settings, respectively. The effect sizes for book ownership were even larger: 0.85 and 1.25 standard deviations in urban and rural settings, respectively. The current study did not use an experimental design, so no cause-effect inferences can be drawn. In particular, given its positive associations with toy ownership, preschool attendance, and home learning activities, it seems likely that book ownership is a marker of wider favorable life circumstances, paralleling stunting as a marker of wider unfavorable life circumstances. Nevertheless, the magnitude of the effects found in the current study justify further exploration of the possibility that sustainable interventions can be designed by capitalizing on the existing household resources and practices that are associated with book ownership and no- or low-cost home learning activities.

### 4.7. Limitations

Several limitations should be considered when interpreting the current findings. First, the measures included in MICS5 had many shortcomings. No information about the dose or quality of children's preschool education or home learning activities was collected; the ECDI has limited sensitivity, and the skills it assesses may not have the same priority in all contexts (89); and no measure of the adequacy of children's recent nutrition was available. Thus, the current findings should be interpreted with caution.

Second, the current analyses included only one covariate, children's age. Many previous studies have included a large number of covariates, most of which relate to adverse life circumstances [e.g., (17, 19)]. The latter approach is inconsistent with the conceptual model adopted in the current research, in which stunting is perceived to be an indicator, and outcome, of adverse life circumstances. In addition, previous research suggests that the relationships between the distal predictors that are commonly included as covariates (such as low maternal education level and family socio-economic status) and children's outcomes are strongly mediated by the proximal factors that were the focus of the current study (preschool attendance and home-based learning activities and resources) (102). Our decision was validated by results showing that the proximal predictor variables fully accounted for urban–rural discrepancies in school readiness scores, without the need to add measures of parental education, wealth or other distal factors. However, greater attention to the interplay between parental education, wealth and other dimensions of children's life circumstances is needed to inform interventions for stunted children. In particular, we know little about the life circumstances of the growing population of poor urban children in many low-and middle-income countries. These children too are at elevated risk of stunting (20).

### 4.8. Policy implications for Côte d'Ivoire

Like many other low-income countries, Côte d'Ivoire has struggled to provide a basic education for its children. In their final year of primary school, fewer than half the students in Côte d'Ivoire have acquired the expected skills in reading and fewer than one-third have acquired the expected skills in mathematics (108). Since the MICS5 data were collected, the Ivorian government has instituted the *Education Sector Plan 2016–2025*, that includes a commitment to improving levels of access and quality of preschool education (109).

Several non-government organizations are supporting these policies. For example, the Jacobs Foundation is co-ordinating a coalition that provides expertise, auspices and evaluates pilot projects, and implements effective programs (https://treccprogram.org/en/what-we-do/clef-elan/). Many of these programs primarily target the parenting skills of rural mothers. The current findings highlight the important support for development provided by household members other than parents, and suggest that their inclusion in interventions may be necessary to achieve lasting changes in children's developmental ecology. In addition, most interventions originate outside the communities in which they are implemented. The current results provide tentative support for a positive deviance approach. They raise the possibility of developing effective interventions that capitalize on, rather than seek to supersede, “home-grown” culturally and contextually appropriate “best practice” in child nutrition and childcare. Other authors have suggested ways in which support for early child development can draw on the existing resources of African communities and the strengths of their cultural traditions (110, 111). Whichever approach is adopted, it is unlikely that interventions will reduce chronic or recurrent child undernutrition unless these are accompanied by long-term solutions to food insecurity and the limited availability of affordable nutritious foods. These require a different set of interventions [e.g., (112)].

## 5. Conclusion

The current findings indicate that stunting has a high prevalence among young children in Côte d'Ivoire, and that it is particularly high in rural areas. Stunting is the result of chronic or recurrent undernutrition. However, many studies have shown that stunting is also associated with a range of other developmental disadvantages. In the current research, stunting was associated with life circumstances characterized by higher exposure to diverse developmental risks, lower likelihood of attending preschool, lower diversity of home learning activities, and access to fewer types of home learning resources. In this context, outcomes for stunted children may be influenced by undernutrition, underlying adverse life circumstances, or the combination of these. The current study focused on the foundational skills children need to make a successful transition to school. The results were not consistent with a perspective in which undernutrition is perceived as having a causal relationship with school readiness. Stunting did not show an independent association with school readiness in either urban or rural settings, and children with severe stunting did not have lower school readiness than those with moderate stunting. In contrast, school readiness was associated with other dimensions of children's life circumstances. Its association with the diversity of home learning activities, and access to a specific home learning resource (children's books or picture books) had effect sizes comparable to, or larger than, those reported for many interventions. Most existing interventions delivered to stunted children are resource-intensive, and therefore have limited sustainability and scalability in low- and middle-income countries. The current findings provide grounds for further exploration of the possibility that effective, low-cost and culturally appropriate interventions can be developed by capitalizing on the existing practices of families that show positive deviance in the provision of informal opportunities for learning. Such interventions may be particularly relevant for attempts to support psychological development and academic achievement. Stunted children will continue to need nutrition interventions to support their health and physical growth.

## Data availability statement

The datasets analyzed for this study can be found in the UNICEF archive of datasets from Multiple Indicator Cluster Survey 5: https://mics.unicef.org/surveys.

## Author contributions

AB and FD contributed to all sections of the manuscript. JR mentored the development of the project and edited the manuscript. VT contributed to the discussion and referencing. AA and ES contributed to the introduction and discussion. All authors contributed to the manuscript and approved the final version.
